# Natalizumab-immunogenicity evaluation in patients with infusion related events or disease exacerbations

**DOI:** 10.3389/fimmu.2023.1242508

**Published:** 2023-08-22

**Authors:** Nicolás Lundahl Ciano-Petersen, Pablo Aliaga-Gaspar, Isaac Hurtado-Guerrero, Virginia Reyes, José Luis Rodriguez-Bada, Eva Rodriguez-Traver, Isabel Brichette-Mieg, Laura Leyva Fernández, Pedro Serrano-Castro, Ana Alonso, Begoña Oliver-Martos

**Affiliations:** ^1^ Neuroimmunology and Neuroinflammation Group, Biomedical Research Institute of Málaga-IBIMA Plataforma Bionand, Hospital Regional Universitario de Málaga, Málaga, Spain; ^2^ Red Andaluza de Investigación Clínica y Traslacional en Neurología (Neuro-RECA), Málaga, Spain; ^3^ Department of Anatomy and Legal Medicine, Neuropsychopharmacology Group, Biomedical Research Institute of Málaga-IBIMA, Faculty of Medicine, University of Malaga, Málaga, Spain; ^4^ Department of Medicine and Dermatology, Faculty of Medicine, University of Málaga, Málaga, Spain; ^5^ Department of Cell Biology, Genetics and Physiology, Physiology Area. Faculty of Science University of Malaga, Málaga, Spain

**Keywords:** multiple sclerosis, natalizumab, immunogenicity, anti-drug antibodies, adverse events, exacerbations

## Abstract

**Introduction:**

Natalizumab is a biologic drug for relapsing-remitting multiple sclerosis that may induce the generation of anti-drug antibodies in some patients. Anti-natalizumab antibodies (ANA) increase the risk of adverse events and reduce efficacy, being useful biomarkers for monitoring treatment response.

**Methods:**

Retrospective observational study including MS patients treated with natalizumab that experienced infusion-related events (IRE) or disease exacerbations (DE). ANA were tested by Elisa including a screening and a confirmation assay. Patients were further classified as transient (one positive result) or persistent (two or more positive results) ANA.

**Results:**

A total of 1251 MS patients were included and 153 (12.3%) had ANA with at least one single point determination, which were more frequent among patients with IRE compared to those with DE (21,6% vs.10.8%) during the first six infusions. Two or more determinations ANA were performed in 184 patients, being 31.5% permanently positive and 7.1% transiently positive. Interestingly, 26.1% of patients that experienced DE had persistent ANA, while 2.6% were transient. In contrast, 43% of patients with IRE had persistent ANA, and 9.3% had transient antibodies. Patients with persistent antibodies had more frequently high levels at the first sampling compared to patients with transient ANA.

**Conclusion:**

Real-world evidence shows that the presence of ANA is behind an important percentage of patients treated with natalizumab that experience IRE, as well as DE but in a lower degree. These findings support the need to systematically evaluate ANA towards a personalized management of these patients to avoid undesired complications.

## 1 Introduction

Biological drugs, including therapeutic monoclonal antibodies (mAb), are synthesized using biotechnology and genetic engineering. The biologics licensed applications approved by the FDA have grown over the years (1), leading to a therapeutic revolution for a wide spectrum of diseases comprising rheumatoid arthritis, multiple sclerosis, or cancer. Biologics are specific and effective drugs; however, additional safety concerns may emerge with patients treated with these drugs (2), as they can be recognized by the immune system as foreign molecules and trigger an immune response in the form of anti-drug antibodies (ADAs).

The presence of ADAs can be related to a loss of efficacy of the drug by neutralizing its biological effects, or enhancing its clearance. In addition, ADAs may also have an impact on its tolerability and safety profile, as they have been associated with serious acute immune adverse events such as anaphylaxis, and cross-reactivity with the endogenous counterpart (3). Therefore, the assessment of ADAs was recommended in clinical practice by the European Medicines Agency, that published a guideline with recommendations to the assessment of the immunogenicity of therapeutic proteins (4).

Natalizumab (TYSABRI®; Biogen Idec and Elan Pharmaceuticals, Inc.) is a monoclonal antibody directed against the α4β1 integrin, also known as very late antigen-4 (VLA-4), as well as α4β7 integrin, which are expressed on lymphocytes and monocytes. Natalizumab acts by reducing lymphocyte trafficking across the endothelia, including central nervous system (CNS) vessels. It was the first mAb approved by the US Food and Drug Administration (FDA) for the treatment of highly active relapsing–remitting Multiple Sclerosis (RRMS) due to its efficacy in clinical trials (3, 5, 6) and in real-world evidence (7). However, some patients produce anti-natalizumab antibodies (ANA), that have been found to reduce natalizumab serum levels, and therefore, they may lead to a loss of efficacy, but also higher rates of infusion-related events (IRE) (5, 8). Its development usually occurs within the first 6 months of treatment (9). In clinical trials, 6% of the patients had persistent ANA whereas 3% had transient antibodies that disappeared on follow-up (10). The occurrence of ANA outside the setting of a clinical trial has reported ranges of positivity between 4.5 and 14%, and a higher percentage of persistent than transient antibodies (9, 11, 12). Although persistent ANA are inversely correlated with serum natalizumab concentration, only high antibody titers are associated with very low or undetectable serum natalizumab concentration (13). Therefore, ANA are one of the most useful biomarkers for monitoring treatment response in patients with RRMS, and physicians should be aware of them in patients who experience disease exacerbation (DE) or IRE, since treatment discontinuation is recommended in patients with persistent ANA according to the technical data sheet of the drug.

The aim of the present study is to describe the presence of ANA in a large cohort of patients with RRMS treated with natalizumab from several hospitals of Spain and Portugal that presented IRE or DE.

## 2 Materials and methods

The research laboratory of the Regional University Hospital of Málaga was an authorized center by Biogen for the determination of ANA for Spain and Portugal from 2007 to 2020. A retrospective analysis was performed including all samples sent to our laboratory to test the presence of ANA during this period. All samples were sent anonymized and for clinical purposes.

All serum samples from the different centers were collected immediately prior to a infusion of natalizumab, following the instructions given in the sample collection sheet. This sheet also included the demographic and clinical features like date of birth, specific data regarding treatment infusions (date of treatment onset, number of infusions, date of last infusion), and the reason for suspicion of antibodies against natalizumab (DE or IRE). DE were defined as clinical or radiological progression. This study was approved by the Institutional Research Ethics Committee (Comisión de Ética de la Investigación Provincial de Málaga) of our center.

### 2.1 Anti-natalizumab antibodies detection

The detection and confirmation of antibodies to Natalizumab in human serum were made by an ELISA developed by BiogenNatalizumab reference standards and high positive and low positive quality control (QC) were also provided by the company. The procedure includes both a screening and a confirmation assay (to demonstrate the specificity of the binding interactions in the antibody/drug complex), on the same plate.

Microtiter plates were coated with natalizumab (0.25 ml/ml) and incubated at room temperature (RT) for 12–28 h. After washing the plate, it was blocked with 200µl of blocking buffer during 1-4 hours at RT. Screening controls (QC1, QC2 and negative controls [NC]) and samples were diluted 1:10 in assay diluent. Competition control and samples were also diluted 1:10 in a final concentration of natalizumab of 100 µg/ml. The presence of a high concentration of natalizumab binds the antibodies, forming an antibody/drug complex that is blocked when added to the wells, therefore, no signal is detected. After 75 minutes at RT, screening controls and samples were added to the blocked plate. Next, biotinylated natalizumab at 1 mg/ml was added and incubated for 1 h. After washing, streptavidin-conjugated horseradish peroxidase (SA-HRP) was added to bind the captured biotinylated natalizumab. Finally, the HRP substrate was dispensed. The ensuing color development reaction was then stopped by the addition of a dilute acid solution. The optical density (OD) was then measured by spectrophotometry and it was considered directly proportional to the amount of ANA present in the serum specimen. The assay was accepted when the controls (QC1/NC, QC2/NC and QC1/QC1C) were within the acceptance criteria established by Biogen Idec Inc. 133 Boston Post RoadWeston, MA 02493, USA. A sample was considered positive when its screening OD was higher than the OD of QC2 and the OD competition/OD screening ratio was lower than 0.5.

This is a qualitative ELISA, however, the set of controls included allows to distinguish samples with low and high levels of antibodies. QC2 establish the positivity threshold, and the median of QC1 was used to establish the cut-off for low and high levels of antibodies. In consequence, samples with low antibody levels were defined as a mean absorbance value (OD) above QC2 and below the QC1 median, while high antibody levels as OD above QC1 median.

### 2.2 Statistical analysis

Patients were classified according to the ANA status in three categories: negative (any positive determination post-baseline), transiently positive (a negative determination after a previous positive result) and permanently positive (two or more positive determinations with a minimum interval of one month between each determination).

Descriptive statistics included means and standard deviations for quantitative variables, and percentages for qualitative variables. The Mann-Withney test was used to compare quantitative variables and the chi-square test for qualitative variables. All reported p-values represented two-tailed tests, with p-values <0.05 being considered statistically significant. The statistical analysis was performed using the IBM SPSS software, version 14.5, and graphs were made with GraphPad Prism version 9.0.2 software.

## 3 Results

### 3.1 Frequency of anti-natalizumab antibodies

**Figure 1:**
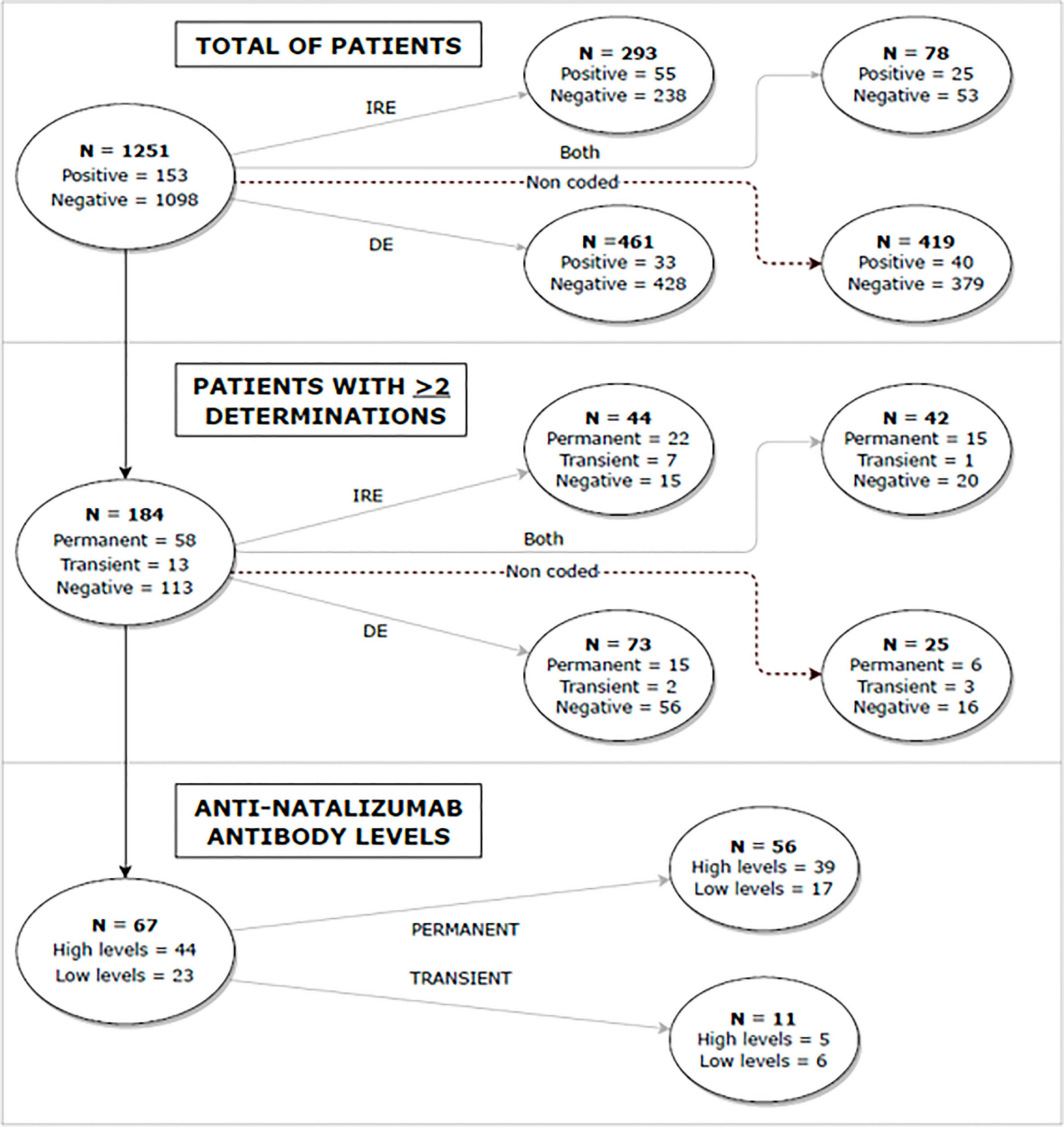
Flow diagram representing the overall design and main findings of the study. DE, disease exacerbations; IRE, infusion-related events.

**Figure 2:**
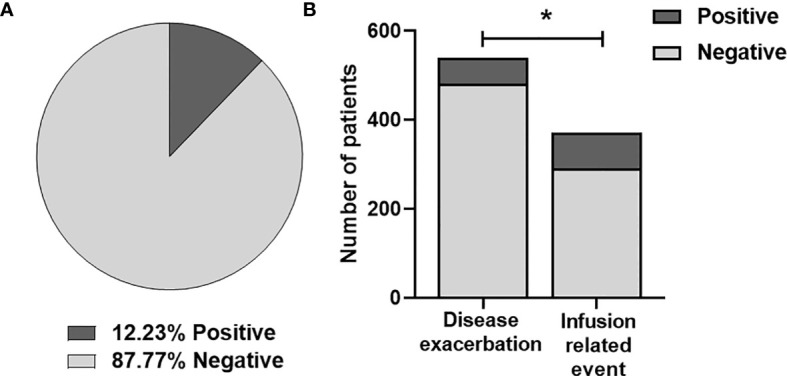
**(A)** Frequency of anti-natalizumab antibodies in all the patients included. **(B)** Analysis of Natalizumab immunogenicity in patients with disease exacerbation and infusion-related events. Differences were analyzed by chi-square test (p-value < 0.001). *, statistically significant.

A total of 1251 patients treated with natalizumab were included in the study (**Figure 1**), of whom 153 (12.33%) had ANA at some point during their follow-up (**Figure 2A**).

If the sample is stratified by the clinical suspicion of developing ANA, 539 (43.1%) patients had DE, but only 58 (10,8%) had ANA. In contrast, among the 371 (29,7%) patients that experienced IRE, 80 (21,6%) had ANA. Interestingly, 78 (6,2%) patients presented both DE and IRE, thus they were included in both groups.

The proportion of patients with ANA was significantly higher in patients with IRE (80 [21,6%] vs. 58 [10.8%]); (p-value < 0.001) (**Figure 2B**).

### 3.2 Frequency of anti-natalizumab antibodies according to the number of infusions

In order to understand the dynamics of ANA, a total of 1380 samples from 1251 patients tested for ANA at different timepoints were included. However, due to the high heterogeneity in the time of sampling intrinsic to the retrospective design of this study, we categorized all samples by 6 month-intervals.

The patients included in this study received a median of 14 infusions of natalizumab (range 1-127).

**Figure 3:**
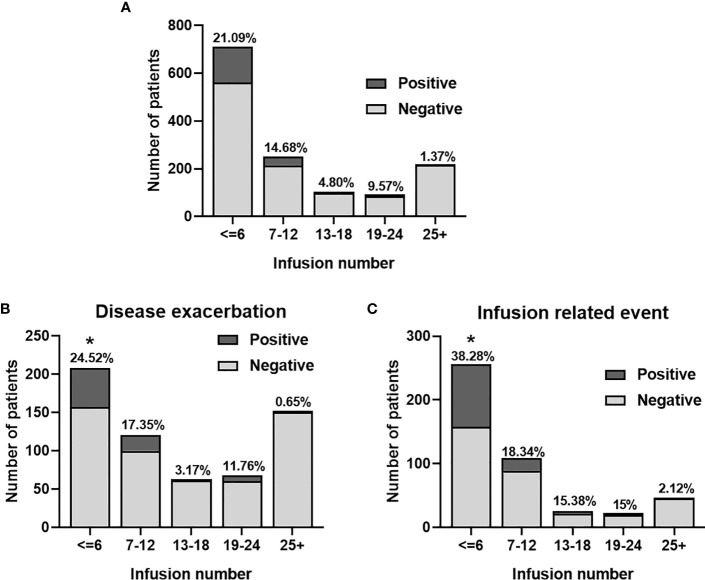
Frequency of anti-natalizumab antibodies according to the number of infusions in the global cohort **(A)**, and according to the reason of testing **(B, C)**. *, statistically significant.

Considering all samples ANA were more frequently identified during the first six infusions (150 [21,1%]) (**Figure 3A**).

Interestingly, ANA were found more frequently during the first six infusions in patients with IRE than in patients with DE (98/256 [38,28%]; vs 51/208 [24,51%]); (p-value = 0.022), as illustrated in **Figures 3B, C**.

### 3.3 Frequency of anti-natalizumab antibodies in patients with multiple determinations

**Figure 4:**
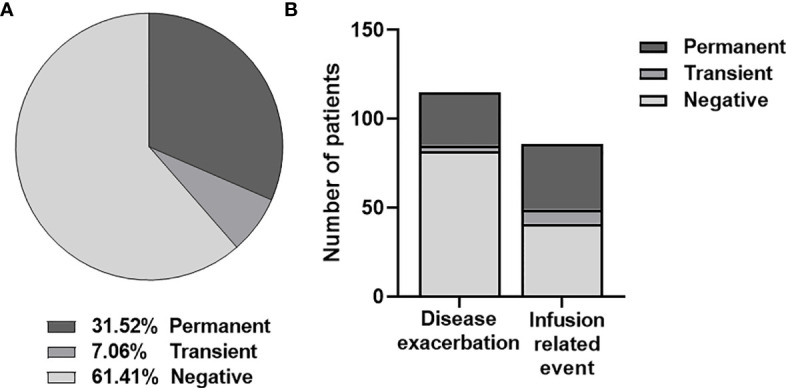
**(A)** Frequency of persistent and transient anti-natalizumab antibodies according to two or more determinations. **(B)** Percentage of persistent and transient anti-natalizumab antibodies in patients with disease exacerbation and infusion-related events.

Among 1251 patients, 184 (14.7%) had two or more determinations of ANA with a median time between determinations of 42 days (range 2-169). Persistent ANA were found in 58 (31.5%) patients, and 13 (7.1%) had transient antibodies (**Figure 4A**). There was no difference between the median time of sampling among patients with persistent and transient antibodies (64 vs 42 days; p>0,05).

If stratifying according to the reason for antibody testing, among the 115 (62.5%) patients that experienced DE, 30 (26.1%) had persistent ANA, while only 3 (2.6%) had transient antibodies. In contrast, out of the 86 (46.7%) patients that presented IRE, 37 (43%) had persistent ANA, and 8 (9.3%) had transient antibodies. On this occasion, 42 (22.8%) patients presented both DE and IRE, so they were included in both groups (**Figure 4B**). Persistent and transient antibodies were found more frequently in patients with IRE compared to patients with DE, although it did not reach statistical significance (p-value = 0.076; p-value=0.051).

### 3.4 Levels of anti-natalizumab antibodies

Samples from patients with two or more determinations were used to evaluate the association between the persistence of ANA and levels of antibodies in the first positive sample. As previously described in the section *Methods*, samples with low antibody levels were defined as a mean absorbance value (OD) above QC2 and below the QC1 median, while high antibody levels as OD above QC1 median.

**Figure 5:**
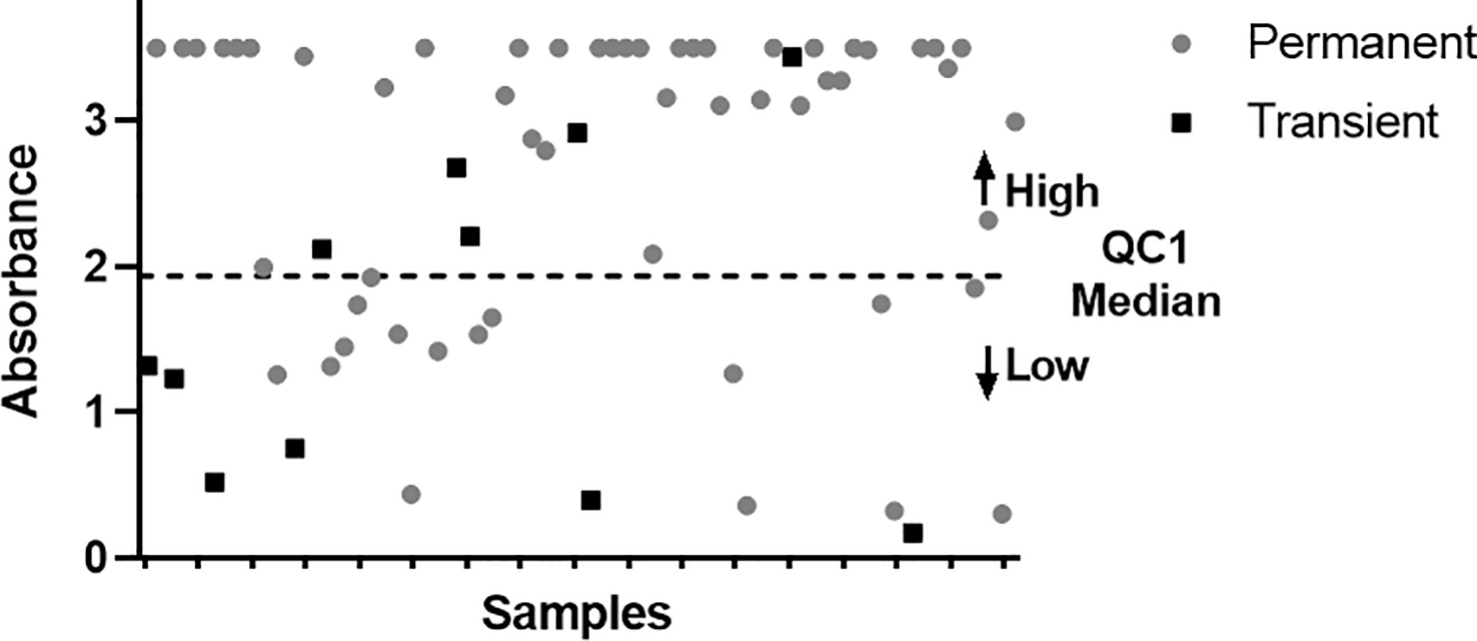
Graphic showing the distribution of persistent (grey circle) and transient patients (black square) with respect to the cut-off (QC1 median). Y axis represents the OD values and X axis the samples.

Among 184 (14.7%) patients with two or more determinations, the levels of ANA in the first sample were available from 67 patients. Accordingly, 56 (83.6%) patients had persistent ANA and 11 (16.4%) had transient antibodies (**Figure 5**). Patients with persistent ANA had more frequently high ANA levels at the first sampling compared to patients with transient ANA (44/56 [78.5%] vs 5/11 [45.5%]); (p-value= 0.02).

## 4 Discussion

In the last decades, the pharmaceutical industry has undergone a major paradigm shift due to the development of novel biologic drugs such as monoclonal antibodies. These drugs are highly specific since they are directed against a specific molecular target; however, their molecular structure can trigger an undesirable immunogenic reaction, that in some cases is driven by ADAs (14, 15). These immunogenic reactions were initially linked to the murine origin of the first monoclonal antibodies. In an attempt to overcome this tolerance issue, novel biotechnology techniques have allowed the development of chimeric, humanized, and human monoclonal antibodies, with the consequent decrease in immunogenicity (16). However, ADAs can appear with all types of monoclonal antibodies, regardless of their nature. Almost 90% of biological drugs approved by the FDA reported the incidence of ADAs, and 49% reported an impact on their efficacy (17). Therefore, it is unquestionable that the assessment of this phenomenon is of utter importance for patients on biological therapies due to the potential impact on safety and efficacy, since it constitutes one of the most useful treatment-response biomarkers despite not being widely used in clinical practice.

Natalizumab is a highly effective biological treatment for RRMS but may trigger an immune response in the form of ADAs, that have been associated with adverse effects or loss of clinical efficacy (5, 8), Therefore, their identification may lead to a shift in their therapeutic management to prevent these undesired events. In the first clinical trials, the incidence of these ADA was 9%, although they disappeared over time in up to 3% of patients, while 6% remained persistent (8). Herein, we evaluated the presence of ANA in a large cohort of patients with DE and IRE after the administration of natalizumab. As expected, our cohort presented overall higher rates of ANA than clinical trials (8) and other studies (9, 11, 12), as well as more frequent persistent ANA, likely explained by the exclusive inclusion of patients with DE and IRE. Interestingly, both persistent and transient ANA were more frequently identified among patients with IRE compared to patients with DE.

Despite finding higher rates of ANA, our results are in concordance with the AFFIRM and the SENTINEL studies regarding the more frequent identification of persistent than transient antibodies, and a higher incidence of ANA among patients with IRE (10). Taken together, these findings suggest that ANA may have a stronger impact on the development of IRE as a consequence of the direct immunologic effects of these antibodies. However, the role of ANA on the development of DE probably differs depending on the time of the appearance. DE occurring in the first months from treatment onset are probably associated with an incomplete effect of the treatment. In contrast, DE occurring after 6 infusions are likely related to a direct impact of ANA in its pharmacokinetic and pharmacodynamics due to a decrease of the drug concentration, although other factors may also play a role.

The pathogenesis underlying the synthesis of ANA is not completely understood. The interplay between factors intrinsic of the patient, the nature of the molecular structure or formulation of the drug, and other unknown factors trigger the immunotolerance breakdown leading to ANA in some patients, however, the reason for their frequent disappearance after an initial synthesis remains unclear (18).

In our previous study, we described that ANA are developed for the first time within the first 6 natalizumab infusions (19). Herein, we observed similar findings, however, due to the high variability in the time of sampling, we were not able to determine the exact dynamics of their synthesis. Interestingly, ANA were more frequently identified within the first 6 infusions among patients with IRE than DE. This finding is in line with the clinical observation of hypersensitivity reactions and infusion reactions (urticaria or rash) to natalizumab generally occurring after the second or third infusion (20) (21), while DE associated with ANA likely occur after 6 or more natalizumab infusions. Thus, our results suggest that the monitorization of ANA is more useful during the first 6 months, especially in case of IRE, while their role after this timepoint is unclear in case of DE unless there is a strict chronological correlation.

Most previous studies used the ELISA developed by Biogen to evaluate ANA (7)- (11). Herein, we also used this analytically validated test to asses them in clinical practice that reports semi-qualitative results. However, antibody levels can be estimated by the intensity of absorbance of the samples compared to a set of quality control samples that determines the threshold for low and high levels of antibodies. Indeed, this estimation has been found to strongly correlate with other quantitative techniques (22). In this study, we observed that patients with persistent ANA during follow-up had more frequently higher levels of antibodies in the first sampling, in line with previous studies reporting that early high titers could accurately predict their persistence (22). However, the validation and standardization of a quantitative method to identify high levels of ANA at the first determination may ease the identification of patients at high risk of developing further side effects.

It’s important to mention that clinicians should be aware that clinical decisions must not be taken considering a single determination of ANA, as treatment withdrawal is only advised if persistent antibodies are found (23), although a first determination with high levels of ANA should warrant attention to the possibility of persistent antibodies. Subsequently, if a second determination performed after at least 6 weeks confirm their persistence, as indicated in the datasheet (23), a careful reassessment of the treatment strategy should be considered to avoid these side-effects, enabling clinicians to decide whether to change treatment in order to avoid loss of efficacy and increase of IRE.

This research study, despite including a large number of patients, has some limitations related to its retrospective design, mainly regarding the heterogenous time of sampling, limited clinical features included in the sample collection sheet, and the uncertainty of the clinical decision after confirming ANA. Another limitation could be the absence of a control group without DE and IRE to directly compare our findings.

In conclusion, this study provides real-world evidence about the development of ANA in patients with highly active MS, proving that these antibodies are behind a non-negligible percentage of patients that experience IRE, but also DE. Furthermore, their levels and persistence in time seems to have an impact in the pathogenesis of these complications. Therefore, the monitorization of ANA at 6 months might lead to a better use of resources, less undesired events, and grants the possibility of applying personalized management to improve outcomes of these patients.

## Data availability statement

The raw data supporting the conclusions of this article will be made available by the authors, without undue reservation.

## Ethics statement

The studies involving humans were approved by Comisión de Ética de la Investigation Provincial de Málaga. The studies were conducted in accordance with the local legislation and institutional requirements. The participants provided their written informed consent to participate in this study.

## Author contributions

NC-P and PA contributed equally to this work and share first authorship. Data collection, data analysis, writing and figures. IH, JR-B: Laboratory Data collection. IB, LL, PS-C: Clinical data collection. AA, BO-M contributed equally to this work and share last authorship. Desing of the study, data analysis, final version of the manuscript. All authors contributed to the article and approved the submitted version.
